# Phonemic verbal fluency task in adults with high-level literacy

**DOI:** 10.1590/S1679-45082016AO3629

**Published:** 2016

**Authors:** Patrícia Romano Opasso, Simone dos Santos Barreto, Karin Zazo Ortiz

**Affiliations:** 1 Escola Paulista de Medicina, Universidade Federal de São Paulo, São Paulo, SP, Brazil.; 2 Universidade Federal Fluminense, Niterói, RJ, Brazil.

**Keywords:** Language tests, Reference values, Adult

## Abstract

**Objective:**

To establish normative parameters for the F-A-S form of the phonemic verbal fluency test, in a population of Brazilian Portuguese speaking adults with high-level literacy.

**Methods:**

The sample comprised 40 male and female volunteers aged 19 to 59 years, and at least 8 years of formal education. Volunteers were first submitted to the Mini-Mental State Examination and the Clock Drawing cognitive screening tests, then to the F-A-S Verbal Phonemic Fluency Test; in this test, examinees were given 60 seconds to generate as many words as possible beginning with each of the three test letters.

**Results:**

The means for number of words beginning the letters F, A and S and for total number of words beginning with either letter generated per minute corresponded to 15.3, 14.4, 13.9 and 43.5, respectively.

**Conclusion:**

Reference values obtained from young adults with high levels of literacy submitted to the F-A-S Verbal Phonemic Fluency Test in this study were similar to those reported in the international literature. These reference values can be used for clinical assessment of language disorder and neuropsychological evaluation.

## INTRODUCTION

In individuals with communication disorders, language assessment can be achieved via formal tests investigating performance in several verbal tasks aimed at differentiating between intact and compromised language processing skills. Language assessment is crucial for appropriate individualized rehabilitation planning.^(1)^ However, assessment procedures must be selected by speech therapists in the light of performance expectations in specific populations.

Verbal fluency tests are commonly used to investigate lexical skills and semantic knowledge in patients with language disorders of neurological origin, such aphasia or other cognitive-linguistic impairments secondary to dementia or head trauma, among others. Cognitive functions, such as attention, long-term memory, mental flexibility, ability to inhibit responses and processing speed can also be assessed using these tests.^(2–4)^

Evocation of verbs and items sharing semantic or phonological criteria are the three major tasks employed in verbal fluency assessment.^(3–7)^ In these tasks, the examinees are requested to name as many words as possible within a given category, typically within a 60 second time frame. Semantic fluency tasks involve words belonging to a predetermined semantic category (*e.g.*, animals, fruits and vegetables, kitchen utensils, supermarket items or given names), while words included in tasks assessing fluency of verbs must belong to this grammatical category. In contrast, in fluency tests with phonological criteria, the examinees are asked to orally produce words beginning with a given letter, F, A and S being the most commonly described in literature and the most commonly used in clinical practice for rehabilitation of patients suffering from acquired neurological disorders.^(3–5,8)^

Sociodemographic and cultural variables, such as formal education, age, sex and nationality may interfere with individual performance on verbal fluency tasks;^(3,4,7–14)^ therefore, studies investigating expected reference values in different populations are vital for accurate assessment.

Normative data for the F-A-S Phonemic Verbal Fluency Test obtained from the elderly population^(3)^ and preliminary data from adults aged under 60 years^(8)^ have been given in Brazilian literature; more recently, a comprehensive verbal fluency trial employing the letter P and including sociodemographic variables such as age and literacy levels has been published.^(15)^

The F-A-S Phonemic Verbal Fluency Test was shown to be sensitive for assessment of functional communication skills in aphasic patients and therefore constitutes a valuable method for assessment and longitudinal follow-up in this group of patients.^(16)^ The F-A-S Phonemic Verbal Fluency Test is a fast, user- friendly speech quality assessment tool; further investigations need to be undertaken to support accurate and reliable application of this test in clinical settings.

Given a significant number of patients requiring assistance due to acquired neurological language disorders are aged under 60 years and that low literacy levels are known to interfere with patient performance in cognitive tasks, studies describing the expected performance of the Brazilian adult population in Phonemic Verbal Fluency Test are required. Studies analyzing the impact of formal education levels are also warranted for comparison of national and international data.

## OBJECTIVE

To establish normative parameters for the F-A-S form of the Phonemic Verbal Fluency Test in a population of native Brazilian Portuguese speakers with high- level literacy.

## METHODS

The sample in this study comprised students, professionals, users of services provided by *Universidade Federal de São Paulo* (UNIFESP), and their family-members or friends. Forty male and female volunteer participants aged between 19 and 59 years, and with a minimum of 8 years of formal education, were randomly recruited through different locations and services within the *Hospital São Paulo* and university facilities, such as classrooms and outpatients clinics. Signed informed consent forms were obtained from all participants.

Individuals with previous or ongoing neurological or psychiatric conditions, non-controlled systemic diseases, self-reported communication disorders, cognitive impairment complaints, history of alcohol abuse or illegal drug use, using psychotropic drugs or presenting with non-corrected visual or auditory deficits that might interfere with performance on tests were excluded. Other exclusion criteria were more than one academic failure reported in school transcript or scores below standard thresholds for the Mini-Mental State Examination (MMSE)^(17)^ and Clock-drawing Test (CDT)^(18)^ cognitive screening.

The MMSE is a cognitive impairment screening test involving verbal and non-verbal tasks related to temporal and spatial orientation, memory, calculation and attention, language and visuoconstructive abilities. Test performance analysis in this study was based on summed task scores; results were then stratified by formal education level. Individuals with 9 or more years of formal education are expected to score at least 28 points in MMSE, according to the criteria established by Brucki et al.^(19)^

For the CDT,^(18)^ the volunteers were given a blank sheet of paper containing a predrawn circle and asked to draw a clock putting in all the numbers and setting hands for 11h10. Scores ranging from 6-10 were attributed according to criteria given by Sunderland et al.,^(18)^ where 10 (highest score) corresponds to drawings with circle and numbers usually intact and hands correctly placed, and 6 (lowest score) to drawings with hands incorrectly placed. Clock drawings with non-intact numbers were also considered in the analysis and 5 (numbers placed at one end of the circle or in reverse order, but hands still present) to 1 (no attempts/rudimentary clock drawing recognized) scores given. Scores of 6 were defined as cut-offs in this study; therefore, volunteers scoring ≤5 were excluded. The CDT was selected due to cognitive screening sensitivity when associated with MMSE.^(20)^

The F-A-S form of the Phonemic Verbal Fluency Test is the most commonly cited in international literature and the most commonly employed in clinical settings.^(3–5,8)^ Also, F, A and S are some of the most common letters in Brazilian Portuguese^(5)^ and introduce similar levels of difficulty to Phonemic Verbal Fluency Test employed in other languages; therefore this test was selected for application in this study.

F-A-S Phonemic Verbal Fluency Test speech samples were recorded using MP4 Philips GoGear Vibe 4GB player.

Participants were interviewed for data collection and those scoring within normality ranges on MMSE and CDT were submitted to F-A-S Phonemic Verbal Fluency Test.

The examinees were instructed to generate words beginning with the test letters F, A and S spelled out loud by the examiner in this order. Examinees were given 60 seconds to name as many words as possible beginning with the first letter; the procedure was then repeated for the two remaining letters.

The participants were informed of inadmissible words (repetitions, proper names or words with different inflection sharing the same root) to be eliminated from the analysis. Application and scoring criteria were derived from Senhorini et al.^(5)^ Instructions were followed by examples using the letter P to illustrate correct and incorrect words. The letter P was selected due to similar frequency of occurrence to F, A and S as the first letter in Brazilian Portuguese words.^(5)^

Words produced by individual volunteers were audio recorded, then transcribed. Volunteer responses were analyzed and scored according to above mentioned criteria. Homophones counted as valid words provided volunteers could explain differences in meaning. Widely used foreign words incorporated to the Brazilian vocabulary and self-corrections were also accepted.

The number of valid words produced for each task was added and the total number of words produced for all three tasks calculated to generate four test scores per volunteer, as follows: total number of words beginning with F; total number of words beginning with A; total number of words beginning with S; total number of words beginning either with F, A, or S. Given the high levels of reliability of verbal fluency task scores reported in literature, inter-rater agreement and test-retest reliability were not estimated in this study.^(21)^

This project was approved by the Research Ethics Committee of *Universidade Federal de São Paulo* (UNIFESP), committee opinion 464.924, CAAE number: 22908313.9.0000.5505.

## RESULTS

This sample comprised 40 individuals, all of whom performed according to expectations in inclusion tests; therefore, there were no losses.

Mean age and length of formal education in this sample corresponded to 37.8 and 14.3 years, with standard deviations - SD of 12.4 and 4.1, respectively. Mean MMSE and CDT scores corresponded to 29.5 points (lowest score, 29 points) and 8.9 points respectively (Table 1).

**Table 1:** Mini-Mental State Examination and Clock Drawing Test scores obtained by adults with high-level literacy evaluated in this study

Results	MMSE	CDT
Mean	29.5	8.9
Median	29.0	9.0
Standard deviation	0.5	1.4
Minimum	29	3
Maximum	30	10
N	40	40
Lower limit for the mean – 1 standard deviation	29.0	7.5
Upper limit for the mean + 1 standard deviation	30.0	10.3
Lower limit for the mean – 2 standard deviations	28.5	6.1
Upper limit for the mean + 2 standard deviations	30.5	11.7

MMSE: Mini-Mental State Examination; CDT: Clock Drawing Test; N: total sample number.

Means and SD for number of words beginning with the letters F, A and S and for total number of words beginning with either letter generated per minute corresponded to 15.3 (4.9), 14.4 (4.1), 13.9 (3.5) and 43.5 (10.9) respectively (Table 2).

**Table 2:** Values obtained by young adults with high-level literacy on the F-A-S Phonemic Verbal Fluency Test

Results	Letter F	Letter A	Letter S	F-A-S
Mean	15.3	14.4	13.9	43.5
Median	15.5	15.0	14.0	44.5
Standard deviation	4.9	4.1	3.5	10.9
Minimum	5	7	7	19
Maximum	27	22	21	67
Lower limit for the mean – 1 standard deviation	10.4	10.3	10.4	32.6
Upper limit for the mean + 1 standard deviation	20.2	18.5	17.3	54.4
Lower limit for the mean – 2 standard deviations	5.5	6.1	7.0	21.8
Upper limit for the mean + 2 standard deviations	25.0	22.6	20.8	65.3

## DISCUSSION

Young adults with high levels of literacy participating in this study achieved similar mean scores on the F-A-S Phonemic Verbal Fluency Test to those reported in international studies. This was the most relevant finding in this trial.

Previous studies showed that the level of education significantly affects performance on verbal and other cognitive tasks (*i.e.*, the higher the level of education, the better the performance).^(22,23)^ Phonemic Verbal Fluency Test, particularly those requiring retrieval of phonological or orthographic information, are known to be even more sensitive to education level interferences.^(15)^ Hence, a population with high levels of literacy was selected for this study.

Normative data for the F-A-S Phonemic Verbal Fluency Test obtained from elderly individuals have been given in Brazilian literature;^(3)^ however, data concerning adults aged under 60 years are scarce and studies with specific focus on the establishment of phonological/ orthographic Phonemic Verbal Fluency Test reference values for adult populations are lacking.

Sixteen participants in this study had between 9 and 11 years of formal education and might have scored 28 points in MMSE; still, the lowest MMSE score documented corresponded to 29 points. Brazilian normative data for the CDT are lacking. However CDT scores in this study were consistent with intact visuoconstructive ability according to criteria given by Sunderland et al.^(18)^ and where therefore thought to be in good cognitive health.

National studies^(8,15)^ aimed at establishing normative data for the phonemic/orthographic Phonemic Verbal Fluency Test have used the letter P on the grounds that this letter in easier for native speakers of Brazilian Portuguese. Although the letter P can be use in clinical practice,^(8)^ authors of this study chose to use the original test format F-A-S to allow comparison with data given in international literature.

Comparative analysis is crucial, particularly for multicenter study planning. Means documented in this trial were similar to those reported in international^(6,10)^ and national studies.^(5,8,24)^ Minimum and maximum numbers of F-A-S words spoken were also similar to values given elsewhere.^(8)^

These findings may be explained by the fact that individuals with high levels of literacy have similar cerebral organization, as shown in studies combining neuroimaging techniques and neuropsychological tasks, which provided significant evidences of the relation between education level and cognition.^(25)^ Such levels of cerebral organization reflect the larger cognitive reserve of highly educated individuals and justify their better performance on neuropsychological tests. In other words, the level of education seems to interfere with cognitive abilities required for good performance on cognitive tasks (*e.g.*, memory, attention, language skills and executive functions). This assumption may explain similar findings across studies involving different populations with high levels of literacy.

### Limitation of the study

This study described mean values, standard deviations and lower and upper limits obtained by healthy young adults with high levels of literacy on the F-A-S Phonemic Verbal Fluency Test. However, cut-offs values for clinical use have not been determined, and the reason would be that the exclusion of results obtained from healthy populations to clinical practice is not recommended.^(26,27)^ In fact, cut-offs suggested sometimes exclude values from normal population. Also, this was a pilot study. Future work involving larger populations are warranted to confirm these preliminary data.

## CONCLUSION

Reference values obtained from young adults with high levels of literacy submitted to the F-A-S Phonemic Verbal Fluency Test were determined in this study; values were similar to those reported in international studies. These reference values can be used for language dysfunction and neuropsychological assessment in clinical settings.
